# *Salmonella*-Based Biorodenticides: Past Applications and Current Contradictions †

**DOI:** 10.3390/ijms232314595

**Published:** 2022-11-23

**Authors:** Anton E. Shikov, Maria E. Belousova, Mikhail V. Belousov, Anton A. Nizhnikov, Kirill S. Antonets

**Affiliations:** 1 Laboratory for Proteomics of Supra-Organismal Systems, All-Russia Research Institute for Agricultural Microbiology (ARRIAM), Pushkin, St. Petersburg 196608, Russia; 2 Faculty of Biology, St. Petersburg State University, St. Petersburg 199034, Russia

**Keywords:** *Salmonella*, pathogens, agriculture, rodents, host specificity, virulence, enterobacteria

## Abstract

The idea of using pathogens to control pests has existed since the end of the 19th century. Enterobacteria from the genus *Salmonella*, discovered at that time, are the causative agents of many serious diseases in mammals often leading to death. Mostly, the strains of *Salmonella* are able to infect a wide spectrum of hosts belonging to vertebrates, but some of them show host restriction. Several strains of these bacteria have been used as biorodenticides due to the host restriction until they were banned in many countries in the second part of the 20th century. The main reason for the ban was their potential pathogenicity for some domestic animals and poultry and the outbreaks of gastroenteritis in humans. Since that time, a lot of data regarding the host specificity and host restriction of different strains of *Salmonella* have been accumulated, and the complexity of the molecular mechanisms affecting it has been uncovered. In this review, we summarize the data regarding the history of studying and application of *Salmonella*-based rodenticides, discuss molecular systems controlling the specificity of *Salmonella* interactions within its multicellular hosts at different stages of infection, and attempt to reconstruct the network of genes and their allelic variants which might affect the host-restriction mechanisms.

## 1. Introduction

Many rodent species cause significant damage to agricultural production. Crop losses per year in Asia are equivalent to an annual diet of 200 million people [1]. Swanepoel et al. reported 5–10% of rice losses in Asia annually. It is worth mentioning that during acute epizootic outbreaks, the damage increased dramatically [2]. In Tanzania, the invasion of *Mastomys natalensis* led to crop losses of up to 48%, with corn loss reaching 80–100% during acute outbreaks. Similarly, different rodents regularly reduce corn yield by 20–30% in Kenya or even provoke a 90% decline in total crop production in some regions of South America. Additional yield reduction can also be caused by rodents consuming grains stored in granaries and warehouses [2]. Moreover, rodents serve as vectors of many infectious diseases in humans and farm animals. Rodents are reservoirs of tularemia, and when the abundance of infected rodents rises, the number of tularemia cases in humans in Northern Europe and Russia increases accordingly [3]. Importantly, rodent species are considered the main factor determining plague spread [4,5]. Not only rodent overpopulation seriously threatens agriculture, but also it endangers public health; thus, both surveillance and rodenticide-based regulation of their population are required.

The repertoire of applied rodenticides can be divided into two major groups according to their mode of action. The first group is represented by anticoagulants disrupting the process of blood clotting. This group includes the following compounds: bromadiolone, chlorophacinone, difethialone, diphacinone, brodifacoum, and warfarin. The second group encompasses several chemical agents exerting diverse toxic effects, namely, zinc phosphide, bromethalin, cholecalciferol, and strychnine [6,7]. Rodenticides are usually applied as bait to be eaten by rodents. However, these baits often attract small birds and mammals, leading to non-target ecological damage. Additional harm to untargeted predators and scavengers can be caused by feeding on the poisoned prey. According to Erickson, over 300 documented cases show modern rodenticides’ effects on birds and other non-target organisms [6].

In order to prevent the adverse impact of conventional rodenticides on the environment and human health, biological agents regulating the rodent population might be seen as a promising alternative [8,9,10]. One such alternative lies in increasing the abundance of avian predators; however, this was not studied properly, especially when dealing with long periods of time [10]. Another approach implies using microbiologically based methods of controlling pest populations. The plethora of bioinsecticides with different modes of action and specificity have shown efficacy and safety for the environment and humans and found wide application in the modern market of biological products [11]. Nevertheless, the use of *Salmonella*-based biorodenticides has not become as widespread due to possible controversies associated with their host-specificity. Regarding that, we here discuss the applicability of *Salmonella* as a biocontrol rodenticidal agent. For this purpose, we primarily focus on the factors determining host specificity, both experimentally validated and bioinformatically predicted.

## 2. The Dawn of *Salmonella*-Based Rodenticides

The genus *Salmonella* belonging to the family *Enterobacteriaceae*, class *Gammaproteobacteria* was named after the American veterinarian Daniel Salmon. In 1885, when studying foodborne disease during the swine cholera epidemic with bacteriologist Theobald Smith, he isolated the causative agent (*Salmonella choleraesuis*, currently, *Salmonella enterica*) [12]. In July 1889, an unknown bacterium killed most of the laboratory mouse population at the Hygienic Institute at Greifswald, Germany. The epizootic was studied by German bacteriologist Friedrich August Johannes Löffler (Loeffler). He isolated and described the bacterium that caused mice’s death and later named it *Bacillus typhi murium* (currently, *Salmonella enterica* subsp. *enterica* serovar *typhimurium*). He then carried out promising experiments on the common voles *Arvicola arvalis* (*Microtus arvalis*) and suggested using it as a rodenticide [13,14]. Subsequently, a mouse bait, namely, pieces of bread inoculated with *Salmonella*, was designed and successfully applied in Thessaly, Greece. Eating the bait caused almost complete death of rodents 9 days after treatment [15]. Obtained results sparked the emergence of commercial bacterial preparations. Pieces of bread moistened with bacterial culture in warm water were scattered over the fields inducing epizootic in 7–14 days. Later on, however, researchers questioned the applicability of Löffler’s bacterium mentioning expensive production, dependence on favorable weather, short expiry date of 8 days maximum, and activity against voles exclusively [16].

At the same time (1890) in France, Jean Danysz isolated the pathogen responsible for the epizootic of the common vole [17,18]. The bacterium was found to be active against rats, mice, and voles. It became the main biological preparation against rats in France before the First World War. The developed biorodenticide was claimed to be harmless to birds, domestic, and other animals. It was implemented to combat South African rinderpest, Australian rabbits, Portuguese oak parasites, and pests of Russian grain as well [19,20].

In Russia, the earliest mentions of bacteria pathogenic to rodents seen as possible population control agents date back to 1893 [21,22]. In 1893, S.S. Merezhkovsky drew attention to the mass death of ground squirrels *Spermophilus musicus* brought for breeding from the Samara Province (Samara Region, Russia). He discovered a rod-shaped bacterium with a wide range of rodenticide activities infecting ground squirrels, domestic mice *Mus musculus domesticus*, wood mice *Apodemus sylvaticus*, and common voles *Arvicola* [22]. Liquid bacterial culture with a volume of 450–800 mL was tested by watering 1 horse, 2 pigs, 2 rams, and 1 calf. Additional experiments were performed on 1 goose, 1 duck, 1 guinea fowl, 2 hens, and 2 turkeys fed with rye flour soaked in 2250 mL of the same liquid culture. [23]. The author reported that the bacterium was safe both for the studied animals and for people who accidentally swallowed the culture liquid while working with the Pasteur pipette [23]. Followed by a detailed description of morphology and biology, the bacterium was termed *Bacillus spermophilinus* according to the isolation resource [24]. In 1897, while studying the microflora of rodents, Boris Issatschenko isolated a more effective rodenticidal bacterium from the corpse of a gray rat which was pathogenic to rats and other small mouse-like rodents such as mice, voles, pies, and hamsters [25,26].

Two bacteria isolated by Issatschenko and Merezhkovsky (*Salmonella enterica* subsp. *enterica* var. Issatschenko and var. Mereshkowsky, respectively) were virulent to some harmful rodent species while other species were non-susceptible (Table 1) [23,24,27].

Until the end of the 20th century, the safety of *Salmonella enterica* subsp. *enterica* var. Issatschenko and var. Mereshkowsky for humans, domestic animals, and livestock was studied extensively [21,23,24,27,28]. Different strains of *Salmonella enterica* subsp. *enterica* var. Issatschenko have been used in the USSR (the Union of Soviet Socialist Republics) for a long time as the grain preparation called “Bactorodencide” [29]. These strains are currently stored in the Russian Collection of Agricultural Microorganisms (RCAM) (http://62.152.67.70/cryobank/login.jsp (accessed on 29 September 2022)). In short, not long after discovering the *Salmonella* genus, certain strains and serovars were marked as effective biorodenticides. Nevertheless, subsequent history revealed that other subspecies and serovars adapted to other hosts and, more importantly, broad-host-range representatives, hampered the progress in developing *Salmonella*-based preparations due to possible unsafety to humans.

## 3. First Clouds in the Use of *Salmonella* as a Rodenticide

With advances in microbiology in the 20th century, the comprehensive taxonomy of the *Salmonella* genus has been developed. After deriving from *E. coli*, two distinct *Salmonella* lineages evolved, namely, *S. enterica* and *S. bongori* [30]. Genetic relatedness and biochemical properties and the six subspecies of *S. enterica* were proposed: *enterica* (I), *salamae* (II), *arizonae* (IIIa), *diarizonae* (IIIb), *houtenae* (IV), and *indica* (VI). Among them, the *enterica* (I) subspecies are often associated with diseases of warm-blooded animals and humans, responsible for almost 99% of salmonellosis cases [31], while others, as well as *S. bongori*, encompass isolates from cold-blooded animals and environmental samples [32,33].

Currently, the *S. enterica* subsp. *enterica* species contains over 2500 serovars, i.e., sets of strains grouped by specific sets of produced antigens [34]. These antigens comprise lipopolysaccharides (LPS) and special secreted proteins, including host colonization factors and effectors allowing bacteria to evade intestinal defense mechanisms [35]. The well-established modern serotyping scheme (Kauffmann–White–Le Minor scheme) is based on the O (polysaccharide) and H (flagellar) antigens [36]. There are 46 O serogroups [37] and 114 H antigens [37].

Notably, particular *S. enterica* serovars selectively infect different vertebrate host species [32]. While some strains of *S. typhimurium* and *S. enteritidis* serovars can invade rodents, others provoke gastroenteritis, sepsis, and fever in humans, poultry, and livestock [38,39,40,41]. Certain *Salmonella* serovars can cause enteric disease only in one specific host, however, a lot of serovars infect a wide range of hosts [35]. This observation raised the question regarding the safety of bacterial-based rodenticides. Broadly speaking, pathogens that are capable of infecting a wide range of host species are called generalists or broad-host-range pathogens. Conversely, pathogens restricted to one host are termed specialists or host-restricted pathogens [42]. The difference between generalists and specialists lies in the molecular basis of the infection process determined by the landscape of bacterial genomes. Generalists appear to rapidly elicit disease symptoms and induce host immune response. Afterward, they are excreted from the body within a few weeks. Specialists, in their turn, may chronically persist in the host body for decades [42]. A distinct subgroup within specialists is presented by so-called host-adapted serovars which cause systemic infection in particular hosts only but can accidentally invade other animals [43]. Host restriction and adaptation are associated with genomic changes, namely, reduced allelic diversity and the absence of some genes responsible for persistence in host cells. The loss of virulence factors during the evolution of intracellular parasitism is thought to be compensated by host proteins [44,45].

It is generally accepted that *S. bongori* and *S. enterica* subspecies II-VII cause diseases in reptiles. However, multiple studies have shown the emergence of new strains pathogenic to mammals. For example, adaptations to humans leading to concomitant enteritis were reported for *S. enterica* subsp. *arizonae* which acquired the ability to infect humans [46] and dogs [47]. Similar observations were done when studying *S. enterica* subsp. *diarizonae* SBO13 and SBO27 isolates [48], and *S. enterica* subsp. *houtenae* strain CFSAN039533 [49]. Strikingly, even for *S. bongori*, thought to be strictly reptile-specific, the capability to invade humans was exhibited. The emerged strain RKS3044 was hypothesized to represent a distinct phylogenetic line that became adapted to humans [50].

*S. enterica* subsp. *enterica* were isolated from a wide range of hosts including cold-blooded animals such as fish, reptiles, amphibians, and warm-blooded mammals and birds [43]. In some cases, evolutionary scenarios of *S. enterica* subsp. *enterica* host-pathogen interactions entailed specialization and subsequent host restriction. Host-restricted serovars include *S. typhi* causing typhoid fever and paratyphoid fever in humans; birds’ pathogens *S. gallinarum*, *S. pullorum*, and *S. hessarek*; abortion-causing ovine-restricted *S. abortusovis*; pigs-restricted *S. typhisuis*, and horse-restricted *S. abortusequi* (Table 2). As stated before, host-adapted serovars can induce systemic infection only in target hosts but they could be present in other groups asymptomatically. This group includes *S. dublin* and *S. choleraesuis* responsible for bacteremia in cattle and pigs, respectively. Serovars *S. typhimurium* and *S. enteritidis* are multi-host adapted. Persistence in various hosts sufficiently increases the number of routes to infect humans entailing acute outbreaks of enteric diseases [51,52]. The effect of enteric infections caused by various *Salmonella* strains on the global healthcare system cannot be overestimated. The total number of invasive infections is over 179 million cases, including 300,000 deaths [49]. Of these, the prevalence of enteric fever attributed to typhoidal serovars reaches over 27 million human infections with more than 200,000 lethal cases [31]. Non-typhoidal salmonellosis poses a comparable threat with 78 to 99 million people affected and 59,000–155,000 deaths each year [31,35]. Notably, 80.3 of these were considered to be of foodborne origin [35]. In the USA only, the Centers for Disease Control and Prevention (CDC) reported 55,961 hospitalizations and 1351 deaths [40], causing a USD 3.66 billion economic loss annually [31]. Of note, evidence shows that the number of outbreaks associated with antibiotic multidrug-resistant strains is steadily increasing [49,51], which could lead to even more losses and fatal cases. In addition to it, a lot of serovars considered animal-bound induce local outbreaks as well. Such emergent pathogens were descended from serotypes *S. dublin* [53] and *S. hessarek* [54]. However, sometimes a generalist serovar can undergo severe genomic changes becoming host-restricted. A vivid example is *S. typhimurium* phage type DT2 which lost infectious potential against other species but pigeons [55].

Therefore, though some *Salmonella* strains are purely host-restricted which supports the possibility of using them as biological control agents, many other strains belong to the generalists’ group or their host restriction was very difficult to determine. Potential human pathogens can occur from serovars thought to be host-restricted. At the same time, generalists occasionally lose the ability to cause infections in multiple hosts. This implies that the border between generalists and specialists is vague and not straightforward. Revealing mechanisms determining these properties requires understanding the molecular drivers of the pathogenesis process.

**Table 2:** Host specificity of *Salmonella* serovars.

*Salmonella enterica* Serovars	Affected Group of Animals	Comments	References
*choleraesuis*	pigs	host-adapted, asymptomatically exist in other animals	[56]
*dublin*	cattle	host-adapted, asymptomatically exist in other animals	[57,58]
*typhi*	humans and higher primates	host-restricted	[59]
*gallinarum*	poultry	host-restricted	[60,61]
*Abortusovis*	ovine	host-restricted	[42]
*typhisuis*	pigs	host-restricted	[43]
Abortusequi	equine	host-restricted	[43]
*typhimurium*	humans, poultry, cattle, pigs, mice	non-host-adapted	[55,62]
*S. typhimurium* phage type DT2	pigeons	host-restricted	[55]
*enteritidis*	humans, poultry, cattle, pigs, mice	non-host-adapted	[42]
*pullorum*	avian	host-restricted	[63]
*hessarek*	avian	host-restricted	[54]

## 4. The Eve of Bacterial Rodenticides

The development of molecular biology at the end of the previous century leads to the understanding that practically all *Salmonella* strains utilize one general pathway of infection. It includes three main stages: (i) luminal colonization, (ii) invasion of epithelial cells, and (iii) bacterial-driven endocytosis or phagocytosis by immune cells. This complex process involves specific virulence factors acting at each step; a significant part of them is encoded by the horizontally acquired Salmonella Pathogenicity Islands (SPIs) [64]. However, other virulence factors are also of great importance, including the virulence plasmid pSLT, adhesins, flagella, fimbriae, and biofilm-associated proteins [12]. Noteworthy, even closely related *Salmonella* strains can harbor strikingly different repertoires of genes encoding virulence factors [65].

At the first stage of infection, *Salmonella* colonizes the intestinal lumen, competing with the normal gut microflora for nutrient sources [66]. Recent studies have demonstrated that *Salmonella* uses the SPI-6-encoded type VI secretion system (T6SS) as the main weapon to prevail over other bacterial species. The T6SS is a multiprotein system consisting of a needle apparatus and effector proteins. *Salmonella* kills commensal gut bacteria in a T6SS-dependent manner using the amidase effector protein causing lysis of target prokaryotic cells [67,68,69]. Surface proteins (especially, flagellins) and LPS produced by *Salmonella* are recognized by pattern recognition receptors of the intestinal epithelium and immune cells launching the inflammation process which is needed for successful lumen colonization by these bacteria since it allows them to grow quicker than the resident gut microbiota [66]. A probable explanation of this effect is that activation of the inflammation pathways provides *Salmonella* with specific respiratory electron acceptor tetrathionate that cannot be utilized by fermenting gut microbiota [70]. Additionally, *Salmonella* cells withstand the influence of antimicrobial peptides produced by the Paneth cells by suppressing peptide expression using the type III protein secretion system (T3SS) [71] that modifies LPS structure to decrease its negative charge, thus reducing the attraction of the positively charged antimicrobial peptides [72].

The attachment of *Salmonella* to host epithelial cells is mediated by a gel-like mucosal layer composed of glycoproteins called mucins [66]. In contrast to several pathogenic bacteria, *Salmonella* does not degrade intestinal mucins but recognizes several mucins as the binding sites [73]. The type III protein secretion systems (T3SS-1 and T3SS-2) encoded by SPI-1 and SPI-2, respectively, are used as key virulence factors [74]. Proteins encoded by genes located in less studied pathogenic islands SPI3 [75], SPI4 [76], and SPI5 [77,78] are also involved in controlling attachment and intestinal colonization. Somewhat similar to T6SS, T3SS represents a sophisticated molecular machine consisting of a needle protein complex and a set of effector proteins and translocases transported via needle from bacteria to eukaryotic cells [79,80]. So-called “effector” proteins of T3SS-1 are used to modulate different molecular processes in host cells via direct injection into them [81]. *Salmonella* possesses an arsenal of effector proteins to colonize different host tissues ad persist there [74]. Using T3SS-1 effectors, *Salmonella* directly and indirectly (through activating host GTPases) induces actin cytoskeleton rearrangement in epithelial cells, provoking membrane deformation [66], and increasing tight junction permeability [82]. These events are required for the internalization of bacteria within a specific compartment called *Salmonella*-containing vacuole (SCV) [81]. After SCV formation, the T3SS-1 effectors inhibit host pro-inflammatory cascades and restore the host cell membrane to the initial state. Not only T3SS-1 effectors but several phage-encoded effectors like SspH2 facilitate the down-regulation of the host pro-inflammatory response [81]. Next, SCV undergoes maturation and positioning facilitating replication of the bacterial cells, their further trafficking to the basolateral cell surface, and entry to the *lamina propria.* Major effector proteins acting at the replication and subsequent stages belong to the T3SS-2 system though several T3SS-1 effectors like SipA, SopB, and SptP are also involved in controlling these stages [81,83].

The third stage can lead to the development of systemic infection, which is limited by bacteria survival in the host immune cells that take up *Salmonella* in the *lamina propria.* The major virulence system providing survival of *Salmonella* in macrophages is the aforementioned T3SS-2 encoded by SPI-2 [84]. Additionally, proteins encoded by SPI-5 [85], SPI-11 [86], SPI-12 [87,88], and phage-encoded proteins [89,90], contribute to the survival of *Salmonella* [90]. Subsequent colonization of individual organs by *Salmonella* is not well studied. Still, this process is known to involve alternative routes, and *Salmonella* is contained within host cells possessing macrophage markers even at the late stage of the infection [91]. The main virulence systems controlling the systemic spread of *Salmonella* are encoded by SPI-13 [92,93] and SPI-16 [64,94].

The close similarity of the infection pathways utilized by different strains of *Salmonella* could hardly provide strict host restriction, particularly considering recombination and horizontal gene transfer, especially if virulence factors-encoding genes are transferred from genomes of broad-host-range strains. Given all this, more careful consideration of the safety of bacterial rodenticides was required.

Several commercial preparations for controlling the rodent population in the UK based on *S. enteritidis* (now *S. enterica* subsp. *enterica*), namely “Liverpool Virus”, “Institut Pasteur Virus”, “Ready Eat Relief Virus”, “Danysz Virus”, “London Virus” and “Satin”, were found to be potentially pathogenic for some domestic animals and poultry [95]. *S. enteritidis* var. *danysz* was associated with outbreaks of gastroenteritis in humans. At least two cases in Denmark in 1928–1930, affecting 52 individuals were reported [95,96]. One case was fatal, the child who ate the bait soaked in bacterial preparation “Ratin” died in two days. After the autopsy, *S. enteritidis* was determined as the cause of death. In another case, three patients cooked baits with “Ratin” at the dinner table, after that, they were hospitalized with typical symptoms of gastroenteritis. Subsequently, *S. enteritidis* var. *danysz* was isolated from the feces of one patient. Importantly, all patients successfully recovered from enteric disease. Later, an outbreak of foodborne infection in Helsinki involving 430 people was reported. The infection was caused by the consumption of milk produced on the farm where a rat “virus” was applied [97]. In the UK in the 1930s and 1940s, several outbreaks of salmonellosis emerged. Disease spread was associated with baits soaked with bacterial rodenticides [98]. In addition, the application of *Salmonella*-based biorodenticides sparked the epizootic of game animals such as hares and hamsters [96]. Due to the aforementioned cases, *Salmonella*-based rodenticides were banned in the US (in the 1920s). Following that, their usage was banned in Germany (in the 1930s) and in the UK (in the 1960s) [98]. Nevertheless, *Salmonella*-based rodenticides like “Biorat” (Labiofam, Cuba) [99] are currently produced and utilized in different countries, mainly in Asia and South America [98,100].

Thus, *Salmonella*-based rodenticides have a long, more than one century history of worldwide application and controversial results being prohibited in several countries and approved in others depending on the particular strains and biopreparations.

## 5. New Horizons: Understanding of the Molecular Diversity of *Salmonella*

**Figure 1:**
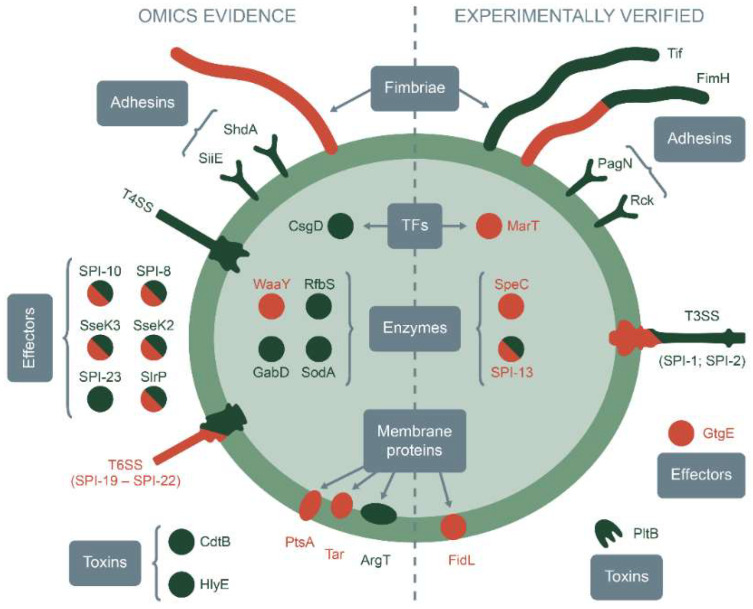
The main proteins and molecular systems involved in the host restriction of *Salmonella* strains. On the left side are the items supported by omics-derived data, and on the right are the ones supported by experimental evidence. Red color denotes the proteins, the absence of which increases the host restriction, and the green color denotes the proteins, the absence of which decreases it. Both colors are used when the absence of the protein might increase or decrease the host restriction depending on the context.

The advances in microbiology, cell biology, and high-throughput genome sequencing in recent decades have opened a new era in our understanding of the diversity of the *Salmonella* genus and allowed us not only to discover the general pathways of infection but also to determine the particular molecular mechanisms of pathogen adaptation to different hosts. Currently, there is a great big body of evidence, which might provide a glimpse of key host specificity factors. Principal approaches to examine such determinants fall into two categories: direct molecular analyses that imply turning off certain genes and tracking colonization patterns for specific hosts and indirect studies mostly presented as characterization of genomic features to reveal dissimilarities in gene content between different serovars, either host-restricted or broad-host-adapted ones (Figure 1). Hence, the following subsections describe both approaches.

### 5.1. Direct Examination of Salmonella Specificity Factors

#### 5.1.1. Adhesion to Host Cells

Probably, the most well-studied *Salmonella* protein enabling bacterial internalization and thus contributing to host specificity patterns is the FimH adhesin forming type 1 fimbriae (T1F) [101]. It has been shown that serovar *S. typhimurium* genomes harbor more than 500 nonsynonymous single-nucleotide polymorphisms (nsSNPs) in FimH-encoding genes. At the same time, the allelic diversity in host-restricted serovars is significantly lower [102]. It is noteworthy that even single amino acid changes may determine the preferred host [102]. For example, V41C and V41G mutations promote specificity to humans in *S. typhi* and *S. choleraesuis* serovars, respectively, but Q67R substitution is associated with porcine infection in *S. typhisuis* and *S. choleraesuis* serovars. Notably, variations in the allelic pool are subjected to positive selection [102]. This mutation-dependent host specificity could lead to the assumption that similar variations in host receptors may determine bacterial adaptation as well. However, the link between receptor-ligand binding and host specificity is not always straightforward. For instance, active FimH variants in host-restricted serovar *S. choleraesuis* (*S*Ch) or/and excessive expression of its receptor, calreticulin (GP2), in porcine cells determined remarkably higher adhesion in contrast to non-active *S*Ch FimH variants, silenced GP2 or unrestricted *S*. *enteritidis* FimH protein with lower adhesion potential [103]. Further studies of the interaction between FimH and GP2 in porcine and human cells showed that different FimH isoforms are associated with unequal invasion rates in human cell lines but not in porcine ones. Moreover, the pervasive potential was independent of GP2 variations in hosts thus implying a tentative GP2-unconnected adhesion mechanism [104]. Nevertheless, ARHGEF26 (Rho Guanine Nucleotide Exchange Factor 26) protein was explicitly related to serovar-host interactions participating in SopB- and SopE-mediated *S. typhi* colonization of human HeLa cells and SopB- and SopE-independent penetration in mouse cells [105]. What is more, avian and mouse Toll-like receptor 5 (TLR5) reacted more actively than human TLR5 on *S*. *typhimurium* flagellin [106]. Vice versa, *S*. *enteritidis* flagellin provoked a similar immune reaction regardless of species [106]. Thereby, unique receptor-ligand interactions can enable bacterial specialization in a particular host. Just like FimH, allelic variations of the adhesin/invasin PagN mediate interactions with host cells promoting *S. typhi*’s ability to be engulfed by human enterocytes via cell adhesion in contrast to *S. typhimurium* [107]. It has been generally accepted that the *Salmonella* serovars’ entry mechanism relies on the type III secretion system (T3SS). In serovars *S. dublin* and *S. typhimurium*, T3SS is obligatory for the internalization process [108]. In this case, *Salmonella* initiates the so-called Trigger mechanism by direct invasion of immune cells via actin polymerization through binding to Arp2/3 complexes [109]. Further investigations revealed independent entry routes involving other proteins: Rck controlling invasion in swarming cell cultures [110] and PagN-based communication with mammal cells in the absence or reduced rate of SPI-1-encoded gene expression [111]. Both proteins initiate the Zipper mechanism via activating tyrosine kinase through its surficial receptor thus recruiting Arp2/3 through a chain of indirect reactions within the signal pathway [109]. However, it was illustrated with scanning electron microscopy, that there are Trigger-like and Zipper-like internalization pathways of *S. enteritidis* into the host without using T3SS and Rck/PagN [112].

#### 5.1.2. Propagation in Macrophages

As the demarcation between virulence factors and host specificity determinants is not always, if ever possible, some mechanisms orchestrate both of these processes. Indeed, host restriction usually evolves together with specificity as was demonstrated for *S. typhi* and *S. paratyphi* serovars [113]. In its turn, this virulence-specificity tandem could be linked with the ability to exist in host immune cells. *S. typhi* invades human dendritic cells (DC) exclusively entailing a blockade of antigen presentation to T-cells and efficient survival yet it fails to replicate in murine cells [114]. Contrarily, specialized *S. typhimurium* bacteria successfully proliferated in murine DC but failed to avoid immune response from human T-cells [114]. On the other hand, *S. typhi* did not succeed to proliferate in mouse macrophages whereas *S. typhimurium* remained in macrophages at 4 h post-infection [115]. In a comparable manner, *S. gallinarum* inhibited the release of pro-inflammatory proteins thus ensuring better survival in avian macrophages, and the same pattern was described for *S*. *dublin* in cattle macrophages. Contrarily, *S*. *typhimurium* failed to invade both macrophages [116]. Recently described *S. rissen* host restriction was associated with the invasion of human macrophages, but the impact on mouse macrophages was significantly lower [117]. The genomic composition of the strain resembled *S*. *typhi* in terms of pathogenic islands SPI-1, SPI-2, and SPI-6 with the latter harboring full *S. typhi* colonization factor (*tcf*) operon [117]. *S*. *typhimurium* Variant Copenhagen Phage Type 99 became pigeon-restricted being cytotoxic for pigeon macrophages whereas three porcine-associated *S*. *typhimurium* strains did not exert a considerable effect on them [118]. A newly described *S. typhimurium* strain MpSTM adapted to sparrows was not pathogenic to mouse cells [119]. Furthermore, the MpSTM strain was unable to replicate in non-phagocytic cells of the host and to form biofilm due to susceptibility to oxidative stress [119]. Such properties were explained by a unique genetic landscape including the BTP1 prophage integration, loss of virulence plasmid, and extensive pseudogenization of genes encoding T3SS-2 effectors (*sseJ*, *steC*, *gogB*, *sseK2*, and *sseK3*), catalase (*katE*), tetrathionate respiration (*ttrB*), and adhesive factors (*lpfD*, *fimH*, *bigA*, *ratB*, *siiC*, and *siiE*) [119].

When studying the inflammation process caused by *S*. *typhimurium* and *S. choleraesuis*, different behavior of human monocytes was found. *S. typhimurium* infection was associated with a smaller rate of cytokine production, especially IL-10, and reduced activity of the JAK/STAT pathway in comparison with *S. choleraesuis* which caused severe immune response and inflammation. In this regard, adapted serotypes tend to cause severe sepsis when infecting the host out of the scope of their specialization, whereas broad-host-range strains usually induce self-limiting enteritis in different animals [120].

Pseudogenization and loss of function sometimes can improve bacterial survival. In *S. typhi*, three genes within the *marT*-*fidL* operon encoded by SPI-3 are turned off, which dramatically affects gene expression in stress conditions. When the respective region from *S. typhimurium* was incorporated into the *S. typhi* genome, a reduced replication rate in monocytes was reported. Notably, the detoxification of hydrogen peroxide was impaired accordingly [121]. Deletion of SPI-13 reduced the infection rate of *S. enteritidis* in streptomycin pre-treated mice, however, the invasion of chicken macrophages remained the same. Therefore, SPI-13 can contribute to host specificity by utilizing monoamines and/or hexuronates in host cells in mice macrophages. Probably, due to the fact that chicken cells are not enriched with monoamines, SPI-13 deletion did not change the intensity of infection [122]. Remarkedly, SPI-13 was discovered when analyzing colonization-disrupting mutations of *S. gallinarum* implicating that, SPI-13 determines adaptation to avian hosts as well [123]. SPI-13 also controlled the *S. enteritidis* invasion of murine macrophages. At the same time, it was nonessential for persistence in human cells [124]. In *S*. *typhi*, SPI-13 is replaced with SPI-8 which, however, was not required for internalization in human macrophages. Interestingly, *S*. *typhi* cells with either SPI-8 deletion or harboring SPI-13 from *S. enteritidis* were incapable of internalization of murine macrophages. Hence, SPI-8 in *S*. *typhi* could be involved in the later stages of the infection while the loss of SPI-13 may contribute to human-bound host restriction [124].

#### 5.1.3. Colonization of Mucosal Tissues

*S. typhimurium* and *S. dublin* colonization of ovine intestinal mucosa induced severe alterations in cellular morphology [125]. Meanwhile, no changes were identified for mucosae infected with serovars *S. abortusovis* and *S. gallinarum*. The number of *S. abortusovis*-infected cells was 10-times lower in comparison with other serotypes, however, after oral inoculation, the consequences were highly similar [125]. The rate of *S. abortusovis* colonization was lowered by the mutational turning off invH located on SPI-1 and incorporating the virulence plasmid had no impact [125]. SPI-1-lacking *S. gallinarum* mutant, a decrease in the invasion rate of avian cells was shown but the mutant strain was still able to persist in macrophages [126]. An opposite pattern was described in SPI-2 mutants: they retained pervasiveness to nonphagocytic cells yet failed to replicate in macrophages [126]. Transmission of *S. typhimurium* lies in the usage of reservoir hosts with concomitant asymptomatic infection. Such interactions between *S. typhimurium* and chickens require SPI-1 and SPI-2 that contribute to the colonization of different organs: SPI-1 maintains persistence in the cecum and spleen, whereas SPI-2 ensures colonization in the spleen [127]. Thus, the host-pathogen interactions determining specificity do not always correlate with host adaptation and virulence and/or the persistence in host cells.

#### 5.1.4. Later Stages of Infection

Specialization could also affect the properties of produced toxins. Experiments with humanized mouse cells revealed cell toxicity for *S. typhi* but not for *S. javiana*. Due to the mutations in PltB within binding sites and *S. javiana*’s inability to be internalized by immune and nerve cells, authors presumed that enteric toxins may contribute to host restriction [128]. Alterations in metabolism during host-pathogen molecular dialogue are thought to be reduced in adapted serovars. Polyamine synthesis protein speC is inactive because of gene deletion in *S. typhi* and *S. gallinarum* serovars. Nonetheless, *S. gallinarum* retained an alternative pathway essential for oral infection which is attenuated in *speB* and *speE* double-mutants lacking the ability to synthesize spermidine [129]. A niche-expanding agent, GtgE that exerts its effect through cleaving Rab-family GTPases thus preventing transport of antimicrobial peptides to *Salmonella*-containing vacuole (SCV) is absent in *S. typhi*. However, adding a vector with *gtgE* from *S*. *typhimurium* permits them to invade non-permissive cells [130,131].

In sum, there is no single determinant or a set of universal markers explaining the host preference of *Salmonella* serovars. It seems that for each host-serovar interaction different adaptations could occur. Despite that, the data mentioned allows for identifying major patterns of host adaptation. These include adhesion proteins and the respective host receptors, the ability to survive in macrophages and form biofilms, enteric toxins, multiple SPIs with pleiotropic effects affecting both virulence and specificity, metabolic adaptations, genome plasticity including allelic diversity and specific mutations, gene gain and loss, pseudogenization and prophages acquisition. Still, the list is surely incomplete, and possible novel insights may stem from comparative genomics and pan-genomic studies.

### 5.2. Indirect Identification of Salmonella Specificity Determinants

#### 5.2.1. Comparative Genomics of Host-Restricted and Broad-Host-Range Serovars

Similar to *fimH* allelic variations, a broad range of substitutions was identified when analyzing 70 selected virulence factors in 500 *S*. *enterica* genomes. A richer allelic pool and an abundance of genes encoding virulence factors were found in non-host-adapted serovars [44]. In host-restricted serovars, the absence of several virulence-determining genes was found, namely, *shdA* and *siiE* in human-constrained *S. typhimurium* ST313 lineage or invasive *S. enteritidis* iNTS strains [44]. Nevertheless, gene pseudogenization is not necessarily associated with the loss of functional proteins. For instance, pseudogenized *shdA* in *S. typhi* is transcribed as a truncated protein [132]. Similar frameshift mutations could encode translated yet shortened proteins whose molecular role is yet to be investigated [133]. On a macroevolutionary scale, gene clusters of fimbrial components (such as *sba*, *sbb*, *sbc*, *sdc*, *sdd*, *sde*, *sdf*, *sdg*, *sdh*, *sdi*, *sdj*, *sdk*, *sdl*, *peh*) have undergone multiple horizontal gene transfer events, duplications, pseudogenization, and gene loss primarily in host-restricted serovars [134]. In serovars with either narrow or broad host range, positive selection in SPI-1 and SPI-2 encoding infection effectors was reported, but the rate of selection pressure was unequal [135]. The important role of SPI-1- and SPI-2-encoded proteins was supported by the observation that 6 genes within these islands were found to be ‘differentially evolved’, i.e., developed during host adaptation. It was confirmed by the distance between genomes based on non-synonymous substitution rate coupled and phylogenetic relationships [136]. Isolates from various sources of the same serotype can harbor different molecular determinants. *S. typhi* samples from fish carried SPI-8 only while isolates from blood and water possessed SPI-8 and SPI-10 [137]. Besides, serovars *S.* Washington, *S. enteritidis*, and *S. paratyphi* A also contained SPI-8 and SPI-10 whilst in *S.* Worthington, *S. dublin*, *S. paratyphi* B, and *S. paratyphi* C genomes both islands were absent [137]. The study of three gene clusters encoding Type VI Secretion System (T6SS), namely, SPI-19, SPI-20, and SPI-21, showed that SPI-19 was present in serovars *S. dublin*, *S.* Weltevreden, *S. agona*, *S. gallinarum*, and *S. enteritidis*, wherein most of the island was removed through internal deletion [67]. SPI-20 and SPI-21 were detected only in *S. enterica* subsp. arizonae (IIIa) serotype 62:z4,z23 [67]. Interestingly, SPI-21 bore a locus encoding VgrG protein similar to S-type pyocins of *Pseudomonas aeruginosa*. SPI-6 T6SS was found in a wide range of *S. enterica* strains but was absent in serovars *S. enteritidis*, *S. gallinarum*, *S. agona*, *S. javiana*, *S. paratyphi* B, *S. virchow*, *S.* IIIa 62:z4,z23:- and *S.* IIIb 61:1,v:1,5,(7). Only two genomes (*S. dublin* and *S.* Weltevreden) contained both SPI-6 and SPI-19. Remarkedly, there were lineages with no T6SS loci (*S. enteritidis*, *S. paratyphi* B, *S. javiana*, *S. virchow*, and *S.* IIIb 61:1,v:1,5,(7)) [67]. Hence, the study concludes that T6SS is not exclusively essential for infection, however, it could be beneficial under certain circumstances for specific hosts and different infection stages.

The comparative genomic study of serovars *S. derby* and *S. mbandaka* infecting pigs/turkeys and cattle/chickens, respectively, demonstrated that the serovars possessed different variations of SPI-6. Only in *S. derby*, SPI-23 was present. Moreover, it lacked certain CRISPR loci and exhibited higher intensity of biofilm formation mediated by the CsgD protein. *S. mbandaka*, in its turn, contained operon encoding components of the D-galactonate dissimilation pathway [138]. The pan-genome analysis focused on ancient *S. paratyphi* C revealed that this zoonotic serovar was evolutionarily separated from cattle-adapted serovars *S. choleraesuis* and *S. typhisuis* and acquired two genetic islands, SPI-6 and SPI-7, accordingly. These islands harbor genes taking part in synthesizing the capsular polysaccharide Vi and ejecting effectors through T6SS, respectively [139]. Adaptive evolution also leads to the emergence of multiple SNPs and INDELs in core genes involved in metabolic pathways, especially glutamate metabolism in *S*. *dublin* [63]. Notably, the regulon PhoP up-regulatory network in *S. typhi* contained three extra proteins compared to *S. typhimurium*. These included HlyE, a pore-forming toxin, STY1499 with an unknown function located in the same operon, and the CdtB toxin [140]. *S. pullorum* genome possessed three regions absent in *S. enteritidis*, one of which was T6SS-encoding SPI-19 and almost 1800 SNPs, including variations in genes encoding for T4SS and type III secretion system effector (T3SE) [141]. Not only did the *S. pullorum* genome face multiple events of genome degradation and pseudogenization, but it also acquired many loci by bacteriophage lysogeny and plasmid transfer, and the latter provided multidrug resistance [142].

#### 5.2.2. Proteomic Studies of *Salmonella* Strains with Different Host Specificity

Proteomic screening of host-restricted (*S. typhi*, *S. paratyphi*) and non-host-adapted serovars (*S. typhimurium*, *S. enteritidis*) demonstrated that differentially produced proteins in the first group were related to the outer membrane, stress, and carbohydrate metabolism [113]. On the other hand, proteins in the second group were associated with cell motility and pathogenesis. The dissimilarities, however, referred to in vitro cultures and thus may not reflect the differences during the infection [113]. In a similar manner, the proteome of host-adapted *S. dublin* in the murine model was enriched with SPI-1-encoded T3SS (Type III secretion system) proteins, adhesion, invasion, and stress response factors, whilst in *S. enteritidis* proteome, anaerobic metabolism enzymes, components of nucleobases biosynthesis and antioxidant system were overrepresented [143]. *S. gallinarum* proteome, in its turn, was enriched with SPI-1 effectors, T-cell inhibitors, HSP90, and the RfbS protein involved in oligosaccharides synthesis [129]. Two-dimensional gel electrophoresis-based screening of 5 distinct serovars revealed distinct isoforms of SodA in *S. typhimurium*, lysine arginine ornithine (LAO)-binding amino acid transporter (ArgT) in *S. pullorum* and Succinate semialdehyde dehydrogenase (GabD) in *S. choleraesuis* [144]. *S. typhimurium* definitive type 2 (DT2) strictly restricted to pigeons contained 22 pseudogenized loci which were unchanged in phylogenetically relative *S. typhimurium* SL1344, DT104, and D23580 strains [145]. In addition, a single deletion in the Tar protein-coding gene reduced bacterial chemotaxis to aspartate in vitro. Finally, transcriptomic profiling of analyzed strains showed differentially expressed transcripts connected to motility and flagellum biosynthesis [145].

#### 5.2.3. Revealing Alterations in Host Preferences

Another approach to predicting tentative host specificity factors lies in studying genomic dynamics in the context of specialization emergence, e.g., switching to a novel host. For example, human-adapted *S. bongori* RKS3044 contained distinct T6SS encoded by SPI-22 resembling T6SS located on SPI-19 in *Salmonella* Subgroup I lineages affecting primarily warm-blooded hosts [50]. Similarly, genes *sseK2*, *sseK3*, and *slrP* were absent in environmental samples of *Salmonella enterica* subsp. *diarizonae* but were found in strains SBO13 and SBO27 that gained the ability to provoke human enteritis [48]. *S.* subsp. *houtenae* str. CFSAN039533 harbored the *tcfA* locus, a fimbriae operon previously observed in *S. typhi*, when compared with *S. typhimurium* [49]. Unspecialized non-typhoidal serotypes causing diarrhea instead of systemic typhoid fever tend to organize biofilms in the form of so-called rdar (red, dry, and rough) morphotype with increased adhesion to abiotic and biotic surfaces, which ensures persistence in the non-host conditions [146]. This biofilm formation is controlled by the key regulator, CsgD [147]. Interestingly, a negative rdar morphotype was formed in *S. typhimurium* D23580 and *S*. *enteritidis* D7795, causing invasive disease in Malawian children, and the morphological changes were induced by either switching off or substantially reducing the *csgD* expression resembling C-terminal shortage-mediated CsgD inactivation observed in *S. typhi* [148]. The case report of in-host adaptation of *S. dublin* to humans revealed rapid genomic rearrangements in the bacterial genome, namely, nonsense indels in genes responsible for carbohydrate transport (*ptsA*), lipopolysaccharide (LPS) biosynthesis (*waaY*), and translation (*tufB*) [53].

Studying genomic loci contributing to host specificity in diverse *Salmonella* strains corroborates the conclusion of the previous subsection. The candidate loci include genes encoding for adhesion factors, metabolic pathways components, and infection effectors. Host specificity could also be determined by SPIs acquisition and genomic decay. Thus, the described genomic and proteomic comparisons provide insights that may serve as a roadmap for further experimental research.

## 6. Conclusions

Through more than a century-long history of *Salmonella* research, the idea of using it as a rodenticide has gone a long way from developing new biocontrol agents and increasing their production to the complete ban in many countries. Considering that humans and rodents are all mammals, that there is a common pathway of infection for all strains of *Salmonella* and the ability of bacteria rapidly to evolve, it seems to be too risky to use *Salmonella*-based rodenticides without a detailed understanding of molecular mechanisms underlying their host specificity and biosafety, which is currently insufficient. Still, we might conclude that host-restricted strains exist, but we know a lot about the complexity of mechanisms regulating that restriction. Many components of cellular systems are involved, covering practically all stages of infection, with the key role of adhesion proteins, secretory systems, toxins, transporters, and some metabolic enzymes (Table 3). Unraveling the mechanisms of host specificity is facing difficulties in determining the borders of this specificity because reliable systems to find out the spectra of possible hosts still do not exist. Deciphering a complex network of genes and their allelic variants, which might affect the host restriction, is important for predicting the properties of new strains of *Salmonella* and might be helpful for a better understanding of their epidemiology and the mechanisms of their spreading.

## Figures and Tables

**Table 1:** Pathogenicity of *Salmonella enterica* subsp. *enterica* var. Issatschenko and var. Mereshkowsky to certain rodents.

Affected Hosts	Non-Susceptible Rodents
House mouse (*Mus musculus*), mound-building mouse (*Mus spicilegus*); harvest mouse (*Micromys minutus*), common vole (*Microtus arvalis*), Brandt’s vole (*Microtus brandti*), Major’s pine vole (*Microtus majori*), field vole (*Microtus agrestis*), narrow-headed vole (*Microtus gregalis*), steppe lemming (*Lagurus lagurus*), bank vole (*Myodes glareolus*), European water vole (*Arvicola terrestris*), gray hamster (*Cricetutus migratorius*), mole vole (*Ellobius talpinus*), social vole (*Microtus socialis*)	Field mouse (*Apodemus agrarius*); wood mouse (*Apodemus silvaticus*), yellow-necked mouse (*Apodemus flavicollis*), hamsters (*Cricetus raddei* and *C. auratus*), forest dormouse (*Dryomys nitedula*)

**Table 3:** Putative and experimentally detected specificity factors of *Salmonella*.

Gene/Locus	Product	Type	Impact *	References
Experimentally-validated specificity factors				
*fimH*	FimH adhesin forming type 1 fimbriae	Fimbral proteins	+/−	[101,102,103]
*tcf* operon	*S. typhi* colonization factor	Fimbral proteins	+	[117]
*pagN*	PagN invasin	Adhesins	+	[107]
*rck*	Rck invasin	Adhesins	+	[109,112]
SPI-1; SPI-2	Type III protein secretion system	Secretion systems	+/−	[108,109,110,111,125,126,127]
*marT*	Putative transcriptional regulator MarT	Transcription factors	−	[121]
*fidL*	Predicted inner membrane protein FidL	Membrane proteins	−	[121]
SPI-13	Putative aromatic monoamines-catabolism enzumes	Enzymes	+/−	[121,122,123,124]
*speC*	Ornithine decarboxylase	Enzymes	−	[129]
*pltB*	Typhoid toxin binding subunit PltB	Toxins	+	[128]
*gtgE*	Secreted cysteine protease GtgE	Effectors	−	[130,131]
Predicted by omics studies specificity determinants				
Gene clusters of fimbrial components (*sba*, *sbb*, *sbc*, *sdc*, *sdd*, *sde*, *sdf*, *sdg*, *sdh*, *sdi*, *sdj*, *sdk*, *sdl*, *peh*)	Fimbriae	Fimbral proteins	−	[134]
*siiE*	Non-fimbrial giant adhesin SiiE	Adhesins	+	[44]
*shdA*	Fibronectin-binding adhesin shdA	Adhesins	+	[44,138]
T4SS-encoding loci	Type IV secretion system	Secretion systems	+	[141]
SPI19–SPI-22	Type VI secretion system	Secretion systems	+/−	[50,67,141]
*csgD*	Major biofilm transcriptional regulator CsgD	Transcription factors	+	[138,147]
*argT*	Lysine arginine ornithine (LAO)-binding amino acid transporter ArgT	Membrane proteins	+	[144]
*tar*	Aspartate receptor for chemotaxis Tar	Membrane proteins	−	[145]
*ptsA*	Carbohydrate phosphoenolpyruvate-dependent transporter	Membrane proteins	−	[53]
*waaY*	LPS-inner core-forming HepII-kinase	Enzymes	−	[53]
*rfbS*	Paratose synthase RfbS	Enzymes	+	[129]
*sodA*	Superoxide dismutase SodA	Enzymes	+	[144]
*gabD*	Succinate semialdehyde dehydrogenase GabD	Enzymes	+	[144]
*cdtB*	Cytolethal distending toxin subunit B	Toxins	+	[140]
*hlyE*	Pore-forming toxin hemolysin E	Toxins	+	[140]
SPI-8	Putative secreted effector	Effectors	+/−	[137]
SPI-10	Putative secreted effector	Effectors	+/−	[137]
SPI-23	Putative secreted effector	Effectors	+	[138]
*sseK2*	Translocated effector protein K2	Effectors	+/-	[48]
*sseK3*	Translocated effector protein K3	Effectors	+/−	[48]
*slrP*	Secreted E3 ubiquitin ligase SlrP	Effectors	+/−	[48]

* Plus (+) encodes specificity determinants whose presence is associated with host restriction. Contrarily, minus (−) denotes factors delineating host preference if they are absent in the genomes. Both plus and minus (+/−) imply that the factors either increase or decrease host restriction in different serovars.

## Data Availability

Not applicable.
